# Epigenetic Impacts of Early Life Stress in Fetal Alcohol Spectrum Disorders Shape the Neurodevelopmental Continuum

**DOI:** 10.3389/fnmol.2021.671891

**Published:** 2021-06-03

**Authors:** Bonnie Alberry, Benjamin I. Laufer, Eric Chater-Diehl, Shiva M. Singh

**Affiliations:** ^1^Department of Biology, Faculty of Science, The University of Western Ontario, London, ON, Canada; ^2^Department of Medical Microbiology and Immunology, School of Medicine, University of California, Davis, Davis, CA, United States; ^3^Genome Center, University of California, Davis, Davis, CA, United States; ^4^MIND Institute, University of California, Davis, Davis, CA, United States; ^5^Genetics and Genome Biology, Research Institute, The Hospital for Sick Children, Toronto, ON, Canada

**Keywords:** fetal alcohol spectrum disorders (FASD), epigenetics, neurodevelopment, prenatal alcohol exposure (PAE), early life stress (ELS), oxidative stress, DNA methylation, clustered protocadherins (Pcdhs)

## Abstract

Neurodevelopment in humans is a long, elaborate, and highly coordinated process involving three trimesters of prenatal development followed by decades of postnatal development and maturation. Throughout this period, the brain is highly sensitive and responsive to the external environment, which may provide a range of inputs leading to positive or negative outcomes. Fetal alcohol spectrum disorders (FASD) result from prenatal alcohol exposure (PAE). Although the molecular mechanisms of FASD are not fully characterized, they involve alterations to the regulation of gene expression *via* epigenetic marks. As in the prenatal stages, the postnatal period of neurodevelopment is also sensitive to environmental inputs. Often this sensitivity is reflected in children facing adverse conditions, such as maternal separation. This exposure to early life stress (ELS) is implicated in the manifestation of various behavioral abnormalities. Most FASD research has focused exclusively on the effect of prenatal ethanol exposure in isolation. Here, we review the research into the effect of prenatal ethanol exposure and ELS, with a focus on the continuum of epigenomic and transcriptomic alterations. Interestingly, a select few experiments have assessed the cumulative effect of prenatal alcohol and postnatal maternal separation stress. Regulatory regions of different sets of genes are affected by both treatments independently, and a unique set of genes are affected by the combination of treatments. Notably, epigenetic and gene expression changes converge at the clustered protocadherin locus and oxidative stress pathway. Functional studies using epigenetic editing may elucidate individual contributions of regulatory regions for hub genes and further profiling efforts may lead to the development of non-invasive methods to identify children at risk. Taken together, the results favor the potential to improve neurodevelopmental outcomes by epigenetic management of children born with FASD using favorable postnatal conditions with or without therapeutic interventions.

## Introduction

Human neurodevelopment involves highly orchestrated molecular processes directed by coordinated changes in gene expression. It begins early in ontogeny with the organization of the neural tube and lasts decades. Mammalian neurodevelopment involves a series of events beginning with neurogenesis, followed by cell migration and differentiation, finishing with synaptogenesis and synaptic rearrangement. Neurogenesis is the emergence of neurons from neural progenitor cells in the ventricular zone of what will become the ventricles. Neurogenesis peaks in humans during gestation and continues for years after birth in specific brain regions (Semple et al., 2013). This process is characterized by the accumulation of DNA methylation and histone post-translational modification (PTMs; Lim et al., 2009; Pereira et al., 2010; Wu et al., 2010). Next, the cells must migrate out of this ventricular zone to form the cortex, a process that peaks during early postnatal development (Sanai et al., 2011). Cell migration is mediated by gene expression events, noncoding RNA, and the removal of histone post-translational modifications (Li et al., 2020). Depending on location, the cells will differentiate into neurons, growing axons and dendrites, ultimately forming synapses with other cells. Synapse formation and proper function are also mediated by the removal of histone post-translational modifications and associated regulation of gene expression (Tang et al., 2017). Following competition for neurotrophic factors, apoptotic cell death occurs in neurons that have failed to establish effective communications. Finally, synaptic rearrangement occurs with the formation of new synapses and pruning of inefficient or unused synapses. Synapse formation and pruning begin after 20 gestational weeks and peaks between 8 months and 4 years of age depending on brain region (Lenroot and Giedd, 2006). These processes continue throughout adolescence and into adulthood, albeit at reduced rates. Most notably, maturation of the prefrontal cortex peaks in adolescence and continues for approximately 25 years in humans (Arain et al., 2013). The orchestration of neurodevelopmental cellular processes is sensitive to the prenatal and postnatal environment over time (Tau and Peterson, 2010). The effect of the environment during development has been recognized in many neurodevelopmental disorders. Unfortunately, nearly 18% of children in the United States have developmental disabilities that may be a result of environmental factors (Zablotsky et al., 2019). For example, exposure to early life stress (ELS) *via* neglect or abuse increases the risk of psychiatric disorders later in life (Kisely et al., 2018). Child maltreatment, including physical abuse, emotional abuse, and neglect is associated with later depression, anxiety, post-traumatic stress disorder (PTSD), drug use, suicide attempts, as well as sexually transmitted infections, and risky sexual behavior (Norman et al., 2012; Kisely et al., 2018).

Many neurodevelopmental disorders have multiple and complex contributors. They result from a combination of biological, psychological, social, and environmental risk factors. A broad range of environmental risks can impact neurodevelopment at different stages. One major environmental contributor is maternal alcohol consumption during pregnancy, which is associated with numerous detrimental outcomes, including stillbirth (Cornman-Homonoff et al., 2012), spontaneous abortion (Kesmodel et al., 2002), premature birth (Sokol et al., 2007), birth defects (O’Leary et al., 2010), and growth delays (Sabra et al., 2018). Specifically, prenatal alcohol exposure (PAE) causes fetal alcohol spectrum disorders (FASD), a common, heterogeneous set of neurodevelopmental disorders that begin *in utero*, manifest during childhood, and last a lifetime. As with many neurodevelopmental disorders, the stages of neurodevelopment are influenced by epigenetic control and disrupted in FASD. In this review article, we discuss the context of a developmental epigenetic continuum that is vulnerable to environmental impacts contributing to the manifestation of FASD.

## Neurodevelopment and Fetal Alcohol Spectrum Disorders (FASD)

FASD is an umbrella term for a collection of impairments, including developmental delays, growth restrictions, physical abnormalities, and behavioral deficits that include intellectual disability (Streissguth and O’Malley, 2000; Sokol et al., 2003; Chudley et al., 2005). FASD manifestation is highly heterogeneous, which may be a result of variable dose and timing, as well as other risk factors. Notably, many individuals with FASD only display behavioral and intellectual disabilities that are not accompanied by any obvious physical abnormalities.

FASD is a serious societal concern. It is the leading preventable cause of intellectual disability in the western world. In Canada, an estimated 10% of pregnant women consume alcohol (Popova et al., 2017), and the prevalence of an FASD diagnosis in Canadian 7 to 9-year-olds is estimated between 2 and 3% (Popova et al., 2018). Similarly, in a cross-sectional study of four communities in the United States, the estimated prevalence of FASD is 1.1–5% (May et al., 2018). Despite societal efforts to raise awareness of the risks, gestational alcohol use persists in North America. Unfortunately, this incidence may have increased in recent years. Of the medical harms identified by a recent increase in alcohol-related emergency room visits, suspected fetal damage including fetal alcohol syndrome rose 2,133.3% between 2003 and 2016 in Ontario, Canada (Myran et al., 2019). Given that this concerning trend may extend to populations beyond Ontario, it does not bode well for a focus on FASD prevention *via* alcohol avoidance (Alberry and Singh, 2019).

FASD remains a costly societal burden throughout an affected individual’s lifetime. Individuals with FASD often suffer from poor judgment, are easily distracted, and have difficulty perceiving social cues (Streissguth et al., 1991). Additionally, they often have poor academic performance, social deficiencies, intellectual disability, as well as early and repeated delinquency (Fast et al., 1999; Fast and Conry, 2004). Beyond personal consequences, the economic burden of FASD in Canada in 2013, including costs due to productivity losses, the correctional system, and health care, was approximately $1.8 billion (Popova et al., 2016). In a comparison of economic costs between the United States, Canada, Sweden, and New Zealand, the annual mean cost per person is from $12,000 to $68,000 US dollars per individual per year (Greenmyer et al., 2018). Individuals with FASD have high rates of physical and mental health comorbidities, and high rates of substance use (Popova et al., 2021). FASD diagnosis is challenging as there are no reliable biomarkers nor effective treatments. Additionally, many disorders are comorbid with FASD, with attention-deficit/hyperactivity disorder occurring in 50% of people with FASD (Weyrauch et al., 2017). It will require a concerted effort to prevent or attenuate the detrimental effect of PAE on the developing brain.

Children born with FASD are often born in an unstable home environment that may include various early life stressors. Children with FASD often enter childcare systems such as foster care or orphanages (Lange et al., 2013). Children with FASD have high rates of early life adversity and are less likely to live with both biological parents compared to children without FASD (Flannigan et al., 2021). Specifically, in a recent cross-sectional study, 75% of individuals with FASD were involved with the child welfare system, and these individuals are more than four times more lately to also have an anxiety disorder (Popova et al., 2021). Unfortunately, children with PAE often have comorbid prenatal and postnatal risk factors, including drug exposures and nutritional deficiencies. In a Canadian report of children with confirmed PAE, 95% had other prenatal substance exposure, with 61% experiencing deprivation as a failure to meet basic needs before 2 years old (Lebel et al., 2019). In a study from Washington State, 74% of children with confirmed PAE were not living with their birth parents, with over four out-of-home placements on average per child (Hemingway et al., 2020). In a recent study from one national medical adoption unit in Israel, 20.2% of children had a known history of PAE, and of these just 22.2% had no discernable abnormalities (Tenenbaum et al., 2020). Furthermore, many children without a known PAE history otherwise fit the FASD diagnostic criteria, supporting the notion that FASD is underdiagnosed.

## Cumulative Impacts: Prenatal Alcohol Exposure (PAE) Followed by Early Life Stress (ELS)

Little is known about how ELS negatively impacts children born with FASD (Price et al., 2017). Following PAE and an ELS, such as abuse or neglect during early development, children are more likely to have impaired speech (Coggins et al., 2007), as well as behavioral deficits including impaired memory and attention (Henry et al., 2007; Koponen et al., 2009, 2013). More recently, research has highlighted how common mental health problems are in youth with PAE. While an FASD diagnosis is associated with increased risk for the development of comorbid neurodevelopmental disorders, additional adverse childhood events further increased the rates of neurodevelopmental disorders in people born with FASD (Kambeitz et al., 2019). PAE alone does not sufficiently predict the rate of later disorders and youth with adverse childhood experiences, specifically out-of-home care, have the highest risk of mental health problems (Koponen et al., 2020). Additionally, children with PAE and subsequent postnatal adversity have different brain structures and symptom profiles than children without added postnatal adversity (Andre et al., 2020). Together, these reports highlight the importance of the postnatal environment in the manifestation of FASD. The postnatal environment may improve or worsen the outcome of a child born with FASD but remains to be established. Research involving humans is challenging and often prohibitive. As a result, animal models have become essential tools for characterizing these environmental impacts on neurodevelopment.

## Modeling FASD Including ELS in Rodents

While animal models are a useful tool to explore molecular mechanisms involved in the development of FASD, few have considered the postnatal environment as a modulator of outcome severity. Given the rate of ELS in individuals with FASD, research is now focused on this interaction. The interaction between PAE and postnatal stress has been explored at different age equivalents, including adolescence (Comeau et al., 2014), and adulthood (Hellemans et al., 2008; Uban et al., 2013; Gangisetty et al., 2014; Lan et al., 2015). Our model of PAE and maternal separation stress found sex-dependent changes in behavior following the combination of treatments, with both treatments resulting in changes in activity and learning deficits (Alberry and Singh, 2016). In a novel open field environment, females are more active than males, but not following PAE. There was also increased exploratory behavior in the open field following PAE. Further, PAE and maternal separation stress both result in overnight hypoactivity in a home cage environment, as well as sex-dependent learning deficits (Alberry and Singh, 2016). In a similar rat model from another group, only the combination of PAE and postnatal stress was sufficient to alter anxiety-like behavior (Biggio et al., 2018). In a separate rat model including PAE and ELS, there is altered social recognition memory and hypothalamic neuropeptide expression (Holman et al., 2021).

ELS reduces serum cytokine levels in control rats, but not following PAE (Raineki et al., 2017). Also, serum levels of C-reactive protein, a marker of inflammation, are higher following PAE and higher still with the combination of PAE and ELS. In the amygdala, PAE reduced CXCL1 and IL-10, further emphasizing the potential role of an immune response in FASD (Raineki et al., 2017). Similarly, in our FASD mouse model that includes ELS, changes in hippocampal gene expression are associated with anxiety-like behavior and treatment, specifically genes important for transcriptional regulation and neurodevelopment (Alberry et al., 2020). In a rat model, only the combination of PAE and ELS results in decreased allopregnanolone, a neuroactive steroid (Biggio et al., 2018). While foot shock stress increases plasma allopregnanolone and corticosterone, this response is exaggerated in animals following PAE and ELS (Biggio et al., 2018). The results discussed here are summarized in Table 1, where models of FASD that include ELS differ in rodent species and strains, exposure paradigms, postnatal stressors, sex, timing of assessment, behaviors studied, and molecular features evaluated. The experimental differences may account for the discrepancies in outcomes described in the literature. The gene expression changes following PAE and ELS accompany comprehensive demonstration that different regimes of PAE result in genome-wide changes in brain gene expression in rodents at different stages of neurodevelopment (Kleiber et al., 2012, 2013; Mantha et al., 2014; Gangisetty et al., 2015; Chastain et al., 2019; Lucia et al., 2019; Ieraci and Herrera, 2020; Shivakumar et al., 2020). As gene expression changes likely underlie changes in behavior, the focus becomes on mechanisms of gene expression regulation throughout neurodevelopment.

**Table 1:** A summary of rodent models of FASD that include PAE as well as a postnatal stressor in the literature.

Animal (strain)	PAE dose (timing)	Stressor (timing)	Test timing (sex)	Morphology, behavior, and molecular results (tissue)	References
Mouse (C57BL/6)	10% ethanol solution *ad libitum* (G0–21)	ELS *via* maternal separation (3 h/day; P2–14)	P25–70 (both sexes)	- PAE and ELS reduce activity - Sex-dependent deficits in learning following PAE and/or ELS	Alberry and Singh (2016)
Mouse (C57BL/6)	10% ethanol solution *ad libitum* (G0–21)	ELS *via* maternal separation (3 h/day; P2–14)	P70 (both sexes)	- PAE followed by ELS alters expression of RNA processing and transcription regulating genes, including *Polr2a* (hippocampus)	Alberry et al. (2020)
Rat (Sprague–Dawley)	1 g/kg intragastric administration of 20% ethanol solution (G17–20)	ELS *via* maternal separation (3 h/day; P3–15)	P60 (males)	- PAE and ELS increase anxiety-like behavior, alcohol consumption - ELS after PAE reduces allopregnanolone in response to footshock - PAE and ELS decrease basal corticosterone (blood)	Biggio et al. (2018)
Rat (Sprague–Dawley)	Liquid diet *ad libitum*, 36% ethanol-derived calories (G1–21)	Chronic mild stress (CMS) 2× daily for 5 days (P38–42)	P38–P42 (males)	- PAE reduces body weight, impairs task switching; CMS disrupts cognitive performance; CMS augments PAE disturbances - CMS increases basal corticosterone (blood)	Comeau et al. (2014)
Rat (Sprague–Dawley)	Liquid diet *ad libitum*, 36% ethanol-derived calories (G1–21)	100 μg/kg intraperitoneal lipopolysaccharide (LPS) injection (P60)	P60 (males)	- PAE increases DNA methylation at *Pomc* and reduces expression; PAE increases MeCP2; PAE increases stress response (Corticosterone, adrenocorticotrophic hormone) - MeCP2-shRNA normalizes PAE disturbances (hypothalamus, plasma)	Gangisetty et al. (2014)
Rat (Sprague–Dawley)	Liquid diet *ad libitum*, 36% ethanol-derived calories (G1–21)	CMS 2× daily for 10 days (P60–90)	P60–90 (both sexes)	- CMS eliminates PAE hyperactivity in females; CMS increases PAE-induced anxiety-like behavior - CMS increases post-testing corticosterone, testosterone, and progesterone (blood)	Hellemans et al. (2008)
Rat (Sprague–Dawley)	Liquid diet *ad libitum*, 36% ethanol-derived calories (G1–21)	ELS *via* limited nesting and bedding (P7–12)	P30 and 45 (both sexes)	- PAE and ELS alter social discrimination depending on sex and age - PAE reduces oxytocin (hypothalamus) - ELS reduces vasopressin in different regions based on age (hypothalamus)	Holman et al. (2021)
Rat (Sprague–Dawley)	Liquid diet *ad libitum*, 36% ethanol-derived calories (G1–21)	CMS 2× daily for 10 days (P60–90)	P60–90 (both sexes)	- CMS increases *Crh* and *Avp* expression in PAE males (paraventricular nucleus) - PAE increases *Crh* expression (amygdala)	Lan et al. (2015)
Rat (Sprague–Dawley)	Liquid diet *ad libitum*, 36% ethanol-derived calories (G1–21)	ELS *via* limited nesting and bedding (P8–12)	P12 (both sexes)	- ELS increases vocalizations, PAE pups less than controls - ELS reduces TNF-α, KC/GRO, and IL-10 in controls; ELS further increases serum CRP after PAE (serum) - PAE reduces KC/GRO and increases IL-10 (amygdala)	Raineki et al. (2017)
Rat (Sprague–Dawley)	Liquid diet *ad libitum*, 36% ethanol-derived calories (G1–21)	Chronic variable stress (CVS) 2× daily (P70–80)	P81 (both sexes)	- CVS reduces basal *Crh* for PAE females (prefrontal cortex, bed nucleus of the stria terminalis (BNST)) and control females (posterior BNST) - CVS after PAE increases basal mineralocorticoid receptor (hippocampus), attenuated reduced dopamine receptor expression (nucleus accumbens, striatum)	Uban et al. (2013)

*G indicates gestational day; P indicates postnatal day*.

## Epigenetics: The Interface Between Genome and Environment

The epigenome plays an essential role in mammalian neurodevelopment by regulating the expression of genes required for development and tissue specificity. Epigenetic marks act in concert to regulate chromatin structure, which consists mainly of DNA and associated proteins. Epigenetic marks that regulate transcription include the covalent modification of DNA in the form of DNA cytosine methylation, the PTM of histone protein tails, and non-coding RNAs (ncRNA) that can serve as scaffolds between DNA and proteins. Also included in the scope of the epigenome are ncRNAs that act at the post-transcriptional level to interfere with the translation of mRNA into proteins, such as microRNAs (miRNAs).

The epigenome is dynamic and functions as a responsive interface between the environment (internal or external) and the genome. It enables prenatal and postnatal events to shape neurodevelopment and related behavioral endophenotypes. At each stage of neurodevelopment, epigenetic players are at work. Neurogenesis relies on intergenic de novo DNA methylation by DNMT3A in neural stem cells to maintain active chromatin states for essential genes (Wu et al., 2010). *TET1* deficiency impairs neurogenesis and leads to the downregulation of neural progenitor proliferation genes (Zhang et al., 2013), indicating the importance of the TET family of enzymes responsible for DNA demethylation. Histone modifications also contribute to neurogenesis, given that a knockout of the H3K4me3 methyltransferase, *Mll1*, fails to activate the neurogenesis regulator *Dlx2* (Lim et al., 2009). Similarly, the histone methyltransferase of polycomb repressive complex 2, EZH2, is critical for maintaining the timing and number of cells produced from progenitor cells (Pereira et al., 2010). Cell migration is regulated by long ncRNA (lncRNA) *via* cis-activation of the lysine demethylase *Kdm2b*, a critical factor in the migration of cortical projection neurons (Li et al., 2020). The histone H3K27 demethylase, lysine demethylase 6a (*Kdm6a*), is critical for appropriate synapse formation and transmission through the regulation of genes such as the neurotransmitter receptor *Htr5b* (Tang et al., 2017).

Importantly, many changes in expression following PAE occur for genes with key epigenetic roles, which include proteins important for reading, writing, and erasing epigenetic modifications as well as related ncRNAs. Here, we discuss epigenetic results involving ncRNA, DNA methylation, and histone PTMs in the context of modeling FASD. The results from models discussed in this section are summarized in Table 2, where animal models of FASD differ in species and strains, exposure paradigms, sex, timing of assessment, and molecular features evaluated. These variations may account for differences reported in the literature.

**Table 2:** Summary of animal models of FASD that investigate miRNAs, DNA methylation, and histone post-translational modifications in the literature.

Animal (strain)	PAE dose (timing)	Assessment timing (sex)	PAE-induced changes in morphology, behavior, and molecular features (tissue)	References
Mouse (C57BL/6)	10% ethanol solution *ad libitum* (G1–21)	P70 (males)	- Altered promoter DNA methylation at transcription regulators and agreement with expression changes for brain function (hippocampus)	Alberry and Singh (2020)
Rat (Sprague–Dawley)	2.5 g/kg intragastric administration of ethanol solution 2× daily (P2–6)	P6 and 90 (both sexes)	- Increased adult microglia and expression of inflammation genes; reduced MeCP2, HDAC1, and SIRT1 protein levels; global DNA hypomethylation, increased H3K9ac (hypothalamus)	Chastain et al. (2019)
Mouse (C57BL/6)	two injections of 2.5g/kg ethanol (P4 and 7)	P70 (males)	- DNA methylation changes at peroxisome biogenesis genes and altered expression of free radical scavenging genes (hippocampus)	Chater-Diehl et al. (2016)
Mouse (C57BL/6)	two injections of 2.5g/kg ethanol (P4 and 7)	P70 (males)	- Increased *Tcf7l2* expression and complementary changes in DNA methylation, H3K4me3 and H3K27me3	Chater-Diehl et al. (2019)
Rat (Fisher-344)	Liquid diet *ad libitum*, 6.7% v/v ethanol (G7–21)	P60 (females)	- Increased pituitary weight - Increased dopamine D2 receptor (D2R) promoter methylation, decreased gene expression and protein level; increased expression of *Dnmt1*, *Dnmt3a*, *Mecp2*, *Hdac2*, *Hdac4*, *G9a* (pituitary)	Gangisetty et al. (2015)
Mouse (C57BL/6 × CAST/Ei)	2.9 g/kg intragastric administration of ethanol solution (G1.5 and 2.5)	E10.5 (both sexes)	- Severe growth delays in placentae and embryos - H19/Igf2 DNA methylation unaffected in embryos - Paternal H19/Igf2 hypomethylation in placentae	Haycock and Ramsay (2009)
Pig-tailed macaque	2.5–4.1 g/kg ethanol, once weekly as nasogastric dose (Gestation weeks 5–24)	5.7–6 months (both sexes)	- Decreased 5-formylcytosine and H3K36me3 (ependyma) - Decreased H3K36me3 (dentate gyrus)	Jarmasz et al. (2019)
Mouse (C57BL/6)	10% ethanol solution *ad libitum* (G0.5–8.5)	P21 (both sexes)	- Growth restriction, craniofacial dysmorphology - Altered Agouti viable yellow (A^vy^) expression	Kaminen-Ahola et al. (2010))
Human (embryonic stem cells)	20- or 50-mM ethanol for 24 or 48 h	24 or 48 h after exposure	- Widespread hypermethylation - Altered gene expression for metabolic processes, oxidative stress, and neuronal properties	Khalid et al. (2014)
Mouse (C57BL/6)	10% ethanol solution *ad libitum* (G0–21) Two 2.5 g/kg ethanol in saline injections (G8 and 11), (G14 and 16), (P4 and 7)	P70 (males)	- Altered DNA methylation at genomic imprinted regions containing ncRNAs depending on exposure; altered expression of genes and their miRNAs and snoRNAs (whole brain)	Laufer et al. (2013)
Mouse (C57BL/6)	6 μL/ml of 95% ethanol in embryo media (44 h beginning G8.5)	E10 (both sexes)	- delayed and reduced growth - Global differential promoter methylation with an inverse relationship with gene expression	Liu et al. (2009)
Rat (Sprague–Dawley)	Liquid diet *ad libitum*, 12.5% v/v ethanol (G0–5)	4–6 months 15–18 months (both sexes)	- Spatial memory deficits only in aged females	Lucia et al. (2019)
			- Anxiety-like phenotype only in young females- Increased *Bdnf*, *Grin2a*, and *Grin2b* expression in aged mice; sex-specific increases in *Dnmt1*, *Dnmt3*, and *Hdac2* (hippocampus)
Rat (Sprague–Dawley)	6.37% ethanol solution *ad libitum* (G1–21)	P1, 8, 15, and 22 (females)	- Persistent changes in DNA methylation in genes involved in dopamine signaling, immune response, blood-brain barrier function	Lussier et al. (2018a)
Mouse (C57BL/6)	10% ethanol solution *ad libitum* (G0.5–8.5)	P28 and 60 (males)	- Brain asymmetry - Altered DNA methylation, miRNA, and target gene expression (hippocampus)	Marjonen et al. (2015)
Mouse (C57BL/6)	2 g/kg ethanol in saline injection (G18.5)	4 h after injection (both sexes)	- Heavy-labeled acetate co-administered with maternal ethanol injection results in the incorporation of acetyl groups in fetal brains	Mews et al. (2019)
Mouse (1C11 cells)	50, 150, or 300 mM ethanol for 2, 4, 6, 8, 16, or 24 h in cell media	2, 4, 6, 8, 16, or 24 h post-exposure	- Dose and timing-dependent Dnmt1 reduction, as well as increased *Dnmt3a*, *Dnmt3b*, and *Dnmt3l* expression; increased DNMT3A levels	Miozzo et al. (2018)
Rat (Sprague–Dawley)	Liquid diet *ad libitum*, 36% ethanol-derived calories (G1–21)	G21 and P55 (both sexes)	- At G21, increased total homocysteine and methionine for the dam (plasma), fetal methionine (plasma), and reduces fetal *Mtr* and *Mat2a* expression (whole brain) - Sex-specific changes in adult gene expression related to one-carbon metabolism (hippocampus) - *Slc6a4* expression is associated with promoter DNA methylation (hypothalamus)	Ngai et al. (2015)
Mouse (C57BL/6)	4% ethanol solution ad libitum (G7–16)	E17 (both sexes)	- Impaired cortical thickness, neuroepithelial proliferation, neuronal migration and maturity - Global DNA hypomethylation (whole brain) - Increased MeCP2 protein levels (forebrain)	Öztürk et al. (2017)
Mouse (C57BL/6)	10% ethanol solution *ad libitum* (G0–21)	E18 (both sexes)	- Sex-specific changes in circRNA expression (whole brain)	Paudel et al. (2020)
Rat (Long-Evans)	4.5 g/kg intragastric ethanol solution (G1–22)	P21 (both sexes)	- Increased *Dnmt1*, *Dnmt3a*, and *Mecp2* expression; increased DNMT activity (hippocampus)	Perkins et al. (2013)
Mouse (C57BL/6)	Two injections of 2.5g/kg ethanol (P7)	P7 (both sexes)	- Increased H3K4me3, *Kmt2e*, and *Casp6* expression (cortex, cerebellum)	Schaffner et al. (2020)
Mouse (C57BL/6)	Injections of 2 or 5g/kg ethanol (P7)	P2–90 (both sexes)	- Increased caspase-3 activity, HDAC1–3 levels (hippocampus, neocortex) - Reduced expression of synaptic plasticity genes - HDAC inhibition before PAE rescues these deficits	Shivakumar et al. (2020)
Mouse (C57BL/6)	60, 120, or 320 mg/dL ethanol in cell media (G12.5 for 5 days)	E18	- Reduced H3K4me3 and H3K27me3 in regulatory regions of neuronal precursor identity genes	Veazey et al. (2013)
			- Expression changes in a subset of genes with altered chromatin marks
Mouse (C57BL/6)	Two injections of 2.9 g/kg ethanol (G7)	G17 (both sexes)	- Altered H3K9me2, H3K9ac, H3K27me3 (whole brain)	Veazey et al. (2015)
Mouse (C57BL/6 × CAST)	80, 160, or 240 mg/dL ethanol in cell media for 2 days	2 days after treatment	- Dose-dependent decrease in *Dnmt1* and *Uhrf1* expression; increased expression of *Tet1* and *Tet2* - Dose and timing-dependent changes in H3K4me3, H3K9ac, H3K27me3, and H3K9me2	Veazey et al. (2017)
Mouse (C57BL/6)	2, 4, or 6 g/kg intragastric administration of ethanol solution (G6–15) Whole embryo culture in 2, 4, or 8 mg/ml ethanol solution (G8.5–10.5)	P35–45 E10.5 E17.5 (both sexes)	- Increased malformations, reduced growth - Reduced activity, impaired learning - Reduced *Hoxa1* expression - 14 differentially expressed miRNAs, miR-10a and miR10b are greatest (*Hoxa1* is a target) - Folic acid co-incubation (0.1 or 1 mmol/L) blocks ethanol teratogenesis, upregulates *Hoxa1*, and reduces miR-10a	Wang et al. (2009)
Zebrafish (AB)	0, 1, or 1.5% ethanol in water (3–24 hpf)	24 hpf 4 and 5 dpf	- Delayed growth, developmental defects - Decreased miR-135a, upregulated *Siah1*, activates p38 MAPK/p53 pathway; increases apoptosis - Overexpression of miR-135a protects against apoptosis and craniofacial defects	Yuan et al. (2020)
Mouse (C57BL/6)	10% ethanol solution *ad libitum* (G0.5–8.5)	P87 (males)	- Increased *Slc17a6* expression, promoter DNA hypomethylation and H3K4me3 enrichment (hippocampus) - Decreased protein product VGLUT2 levels, potentially through miRNA action (hippocampus)	Zhang et al. (2015)
Rat (Sprague–Dawley)	Neural stem cells treated with 400 mg/dL ethanol for 6 h	24 and 48 h after treatment (females)	- Reduced migration, neuronal formation, and growth processes - Impaired DNA methylation changes at genes important for neural development and neuronal receptors - Impaired neuronal differentiation	Zhou et al. (2011)

*G indicates gestational day; E indicates embryonic day; P indicates postnatal day; hpf indicates hours post-fertilization; dpf indicates days post-fertilization*.

### Noncoding RNA

Cellular phenotypic outcomes are often the result of inter-related epigenetic mechanisms. For example, noncoding RNAs are important for gene expression regulation at the transcriptional and post-transcriptional levels. They include long non-coding RNAs (lncRNAs; Lee, 2012), microRNAs (miRNAs; Chuang and Jones, 2007), short interfering RNAs (siRNAs), and piwi-interacting RNAs (piRNAs; Holoch and Moazed, 2015). Individual miRNAs also have critical roles in neurodevelopment, particularly for neurogenesis and cell fate specification (Coolen and Bally-Cuif, 2009). More specifically, MeCP2-regulated miRNAs influence neurogenesis and neuronal migration (Mellios et al., 2018). Specifically, in the context of this review, many ncRNAs are environmentally responsive. Interestingly. Circular RNAs (circRNAs) have also been implicated as differentially expressed in the brain in a PAE rat model (Paudel et al., 2020).

The noncoding RNA research in FASD has been focused on the relationship between microRNAs and their mRNA gene targets. Following PAE, genomically imprinted clusters of miRNAs and small nucleolar RNAs (snoRNAs) are deregulated in the adult mouse brain (Laufer et al., 2013). The miRNAs and their predicted mRNA targets are reciprocally differentially expressed and associated with neurodevelopmental events. In a trimester one-equivalent exposure model, microRNAs important for cell state and dendritic spines in the hippocampus are differentially expressed following PAE (Marjonen et al., 2015). In a rat model, third trimester-equivalent ethanol exposure results in increased variance in miRNA expression and increased expression of *miR-200c*, a miRNA important for neurogenesis (Balaraman et al., 2017). In zebrafish, miR-135a is downregulated following ethanol treatment, and its overexpression reduces the associated apoptosis in neural crest cells, growth restrictions, and craniofacial defects (Yuan et al., 2020).

Profiling of miRNA following PAE also holds translational potential. In a recent rat model of PAE, the composition of exosomal RNA in the amniotic fluid was altered following maternal ethanol exposure, with some putative functional roles for differentially expressed miRNAs on stem cell regulation (Tavanasefat et al., 2020). Also, infant circulating extracellular miRNA are predictive of PAE-induced growth restriction and cognitive impairment (Mahnke et al., 2021). Finally, differential miRNA and target gene expression resulting from PAE can be reversed by folic acid supplementation, which is notable given that folic acid is involved in establishing DNA methylation (Wang et al., 2009).

### DNA Methylation

DNA cytosine methylation is a strong candidate for a biological mechanism of action for PAE because ethanol impairs folate transport to the developing fetus (Hutson et al., 2012), a key methyl donor important for DNA methylation. PAE in a mouse model results in changes in expression of the DNA methylation-dependent metastable epiallele, *Agouti viable yellow* (*A^vy^*), indicating that DNA methylation changes occur after PAE (Kaminen-Ahola et al., 2010).

In several studies, PAE alters the expression of DNA methyltransferases, the writers of DNA methylation. In a rat model, PAE results in increased gene expression of DNA methyltransferases, *Dnmt1* and *Dnmt3a*, alongside increased DNMT protein activity (Perkins et al., 2013). In a murine cell culture model, there is a dose-dependent increase in *Dnmt3a*, *Dnmt3b*, and *Dnmt3l* expression, with increased DNMT3A protein abundance (Miozzo et al., 2018). While others have found dose-dependent decreased expression of *Dnmt1* (Veazey et al., 2017). Moreover, increased gene expression of *Dnmt1* and *Dnmt3a* in male rat hippocampus following early PAE (Lucia et al., 2019) or continuous PAE (Gangisetty et al., 2015) has been observed.

Other DNA methylation genes have been implicated in FASD. In rodent models, PAE results in increased expression of the DNA methylation reader methyl CpG binding protein 2 (*Mecp2*) at both the gene (Perkins et al., 2013; Gangisetty et al., 2015) and protein level (Öztürk et al., 2017; Chastain et al., 2019). In a mouse embryonic stem cell model, increased expression of DNA methylation erasers (demethylation genes) *Tet1* and *Tet2* occurs following ethanol treatment (Veazey et al., 2017).

PAE is also known to alter methylation at genes that undergo genomic imprinting, which typically have distinct DNA methylation profiles at regulatory elements for each allele that allow for a parent-of-origin specific gene expression. Several studies regarding DNA methylation have focused attention on a specific imprinted locus in the genome, insulin-like growth factor 2, IGF2/H19. H19 is a long noncoding RNA only transcribed from the maternal allele, while the paternal H19 is normally methylated at the promoter and not expressed. Paternal H19 allele methylation allows for the expression of paternal IGF2, which is not typically maternally expressed (DeChiara et al., 1991). An examination of genome-wide promoter DNA methylation identified hypermethylation at this locus in adult mouse brains following PAE *via* continuous preference drinking (Laufer et al., 2013). Also, targeted bisulfite sequencing of this locus in placentae revealed hypomethylation of the paternal allele in mid-gestation mouse hybrid embryos following pre-implantation alcohol exposure (Haycock and Ramsay, 2009). Furthermore, a genome-wide assay of CpG methylation identified hypomethylation at this locus in the saliva of young children born with FASD (Portales-Casamar et al., 2016). In addition, a polymorphism at this locus is associated with placental DNA methylation and head circumference following PAE (Marjonen et al., 2017). The differences between epigenetic alterations at this locus may be attributed to variations in study design across experiments.

Gene promoter DNA methylation changes following ethanol exposure have been reported in several models. Following ethanol treatment of rat neural stem cells, there is promoter hypermethylation of the chromatin remodeling complex member SWI/SNF related, matrix associated, actin dependent regulator of chromatin, subfamily A, member 2 (*Smarca2*), together with adhesion and polarity genes DiGeorge syndrome critical region gene 2 (*Dgcr2*), and Par-6 family cell polarity regulator alpha (*Pard6a*, Zhou et al., 2011). Conversely, transcription factors cut like homeobox 2 (*Cux2*), POU class 4 homeobox 3 (*Pou4f3*), and SRY-box transcription factor 7 (*Sox7*), are each hypomethylated following ethanol treatment (Zhou et al., 2011). Each of these genes is also critical for neurodevelopment. In a trimester one-equivalent model, there are corresponding changes in DNA methylation status at candidate genes with altered gene expression in the hippocampus, but also in peripheral tissue (Marjonen et al., 2015). Similarly, decreased hypothalamic serotonin transporter (*Slc6a4*) mRNA following PAE was accompanied by increased promoter methylation (Ngai et al., 2015), and increased vesicular glutamate transporter (*Slc17a6*) alongside decreased promoter methylation (Zhang et al., 2015). Binge-like trimester three-equivalent ethanol exposure results in altered promoter DNA methylation, particularly for oxidative stress and peroxisome biogenesis genes (Chater-Diehl et al., 2016).

In our mouse model of continuous PAE, we found altered promoter methylation at genes important for transcriptional regulation. Specifically, PAE led to decreased promoter methylation of polycomb repressive complex 1 members, including chromobox 4 and 8 (*Cbx4*, *Cbx8*) polycomb group ring finger 6 (*Pcgf6*), and ring finger protein 1 (*Ring1*), as well as transcription factor II D complex members general transcription factor II A, 2 (*Gtf2a2*), and TATA-box binding protein associated factors 8, 10, and 11 (*Taf8*, *Taf10*, *Taf11*; Alberry and Singh, 2020). Additionally, there is decreased promoter methylation of nine members of the RNA polymerase II transcription factor complex, including the aforementioned *Gtf2a2*, *Taf8*, *Taf10*, *Taf11*, as well as pygopus 2 (*Pygo2*), nuclear transcription factor-Y gamma (*Nfyc*), peroxisome proliferator activator receptor delta (*Ppard*), and nuclear receptor subfamily 1, group H, member 4 and group D, member 2 (*Nr1h4*, *Nr1d2*; Alberry and Singh, 2020).

Global changes and regional genome-wide differences in DNA methylation following PAE have repeatedly been reported in rodents (Liu et al., 2009; Laufer et al., 2013; Öztürk et al., 2017; Lussier et al., 2018a), human embryonic stem cells (Khalid et al., 2014), and nonhuman primate brain (Jarmasz et al., 2019). In human post-mortem brain samples, reduced DNA methylation in the CA1 region of the hippocampus is associated with PAE (Jarmasz et al., 2019).

In terms of translation potential, research aimed at finding a specific DNA methylation signature in peripheral tissue for FASD has had limited success identifying sites that discriminate children with an FASD diagnosis from those without (Laufer et al., 2015; Portales-Casamar et al., 2016; Lussier et al., 2018b). Also, DNA methylation profiling of blood from children with FASD was able to identify sites related to FASD sub-phenotypes (Cobben et al., 2019), although a strong profile was not observed in cord blood (Sharp et al., 2018). While not yet specific or accurate enough for diagnosis likely due to a combination of effects from genotype, the tissue of origin, and other exposures, these studies suggest the potential for future biomarker discovery. As detection technology and knowledge of DNA methylation states and cofactors improve, there will be improvements in the precision of biomarker detection. Interestingly, while research has established genome-wide DNA methylation profiles as epigenetic clocks for biological aging, there appears to be accelerated epigenetic aging in individuals with FASD (Okazaki et al., 2020).

Using a mouse model combining PAE and ELS, we found different sets of genes with altered promoter methylation following prenatal ethanol exposure, postnatal maternal separation stress, and the combination of treatments (Alberry and Singh, 2020). The combination of PAE and ELS results in decreased promoter methylation of genes critical for neuronal migration, including sema domain, transmembrane domain (TM), and cytoplasmic domain, (semaphorin) 6A (*Sema6a*), unc-51-like kinase 4 (*Ulk4*), disabled 2 interacting protein (*Dab2ip*), and rho/rac guanine nucleotide exchange factor (GEF) 2 (*Arhgef2*). Conversely, the combination of PAE and ELS led to increased promoter methylation of genes important for immune response, including 21 members of the cytokine-cytokine receptor interaction KEGG pathway (Alberry and Singh, 2020). While there is a minimal correspondence between promoter DNA methylation and gene expression, genes involved are critical for brain development and function. These results argue that while promoter methylation and reciprocal alterations to gene expression persist following PAE and ELS, the mechanisms are complex. Gene expression is representative of transient states occurring at that time, while DNA methylation is also reflective of past events and the priming of future ones. Furthermore, some gene expression profiles require activation by specific events. Finally, this discrepancy also suggests that other epigenetic modifications contribute to the lasting changes in gene expression and ultimately the behavioral deficits that occur in FASD, and these include histone PTMs.

### Histone Post-translational Modifications (PTMs)

Histone PTMs are another strong candidate for the mechanism of action following PAE (Chater-Diehl et al., 2017). Modifications to histone tails at specific genomic loci are the foundation of epigenetic mechanisms, with a wide variety of known potential modifications (Allis and Jenuwein, 2016). While modifications can include methylation that is dependent on the same methyl source as DNA methylation, histone acetylation is another common modification. In pregnant mice, exposure to isotope-labeled ethanol results in labeled acetyl groups being incorporated as histone acetylation in the gestating fetal brains (Mews et al., 2019).

PAE is often associated with altered expression of histone modifiers, including deacetylases and methyltransferases, and genome-wide changes in the abundance of modifications. Early embryonic PAE leads to increased *Hdac2* expression in rat hippocampus (Lucia et al., 2019), and continuous PAE results in its increase in female pituitary along with increased *Hdac4* and *G9a* (Gangisetty et al., 2015). Binge-like trimester three-equivalent PAE in rats leads to increased H3 lysine 9 acetylation (H3K9ac) and decreased protein levels of HDAC1 and SIRT1 in the hypothalamus (Chastain et al., 2019), alongside changes in other modifications that may correlate with the phenotype (Veazey et al., 2015). In mice, PAE is associated with increased H3 lysine 4 trimethylation (H3K4me3) levels and increased expression of the chromatin modification gene lysine (K)-specific methyltransferase 2E, *Kmt2e* (Schaffner et al., 2020). It also leads to increased levels of chromatin modifiers, including histone deacetylases (HDAC1-3), and reduced histone 3 (H3) and histone 4 (H4) acetylation in the hippocampus and neocortex (Shivakumar et al., 2020). Furthermore, decreased hippocampal and temporal lobe ependyma H3K36me3 has been observed in a nonhuman primate model of FASD (Jarmasz et al., 2019). In human post-mortem brain samples, PAE is associated with region-specific decreases in H3K4me3, H3K9ac, H3K27ac, H4K12ac, and H4K16ac (Jarmasz et al., 2019).

Altered histone PTMs at specific genes are regularly reported in the literature. In a neurosphere cell culture model, Homeobox genes important for neurogenesis have altered histone H3K4 or H3K27 trimethylation following ethanol treatment (Veazey et al., 2013). While there are often changes in histone PTMs at specific genes, these do not regularly translate into altered expression (Veazey et al., 2017). Following binge-like trimester three-equivalent ethanol exposure, many genes have been implicated by regional differences in H3K4 and H3K27 methylation, particularly increased H3K4 methylation and decreased H3K27 methylation (Chater-Diehl et al., 2016). Interestingly, the Wnt transcription factor *Tcf7l2* is differentially expressed and also has complementary changes in DNA methylation and histone modifications in this model (Chater-Diehl et al., 2016, 2019). These results suggest changes in gene-specific expression in response to PAE may be the result of a variety of epigenetic mechanisms. Although DNA methylation, non-coding RNAs, and histone PTMs have all been implicated, their inter-relationship is not known. The results summarized however emphasize the effect of alcohol on neurodevelopment is likely realized by a combination of epigenetic alterations. Some of these effects are genome-wide, while others represent relatively focused effects. As examples, we will discuss two specific observations involving clustered protocadherins and genes involved in oxidative stress.

## Clustered Protocadherins in FASD and ELS

**Figure 1:**
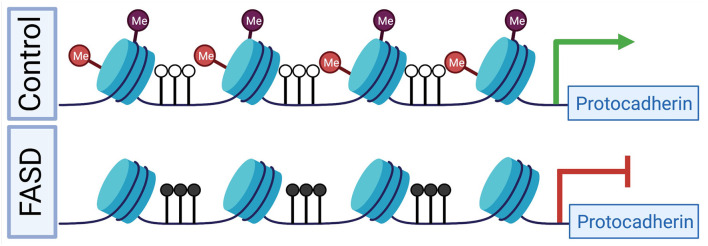
A putative mechanism for differential epigenetic and transcriptomic profiles at the clustered protocadherin locus in fetal alcohol spectrum disorders (FASD). During development in healthy controls, the locus is marked by bivalent histone PTMs, which prevent DNA hypermethylation and poises genes for high expression levels. In FASD, prenatal alcohol exposure (PAE) results in the depletion of bivalent histone PTMs, which leads to DNA hypermethylation and reduced gene expression. Red lollipops represent H3K27me3, purple lollipops represent H3K4me3, black lollipops represent DNA hypermethylation, and white lollipops represent DNA hypomethylation. The green arrow represents normal gene expression, while the red inhibitor shape indicated reduced gene expression. Figure 1 was created with BioRender.com.

Protocadherins are a group of cell-cell adhesion molecules that are highly expressed in the brain and are involved in establishing single-cell neuronal identity. Many of these protocadherins are encoded in a large genomic locus that consists of three multi-gene clusters (Chen and Maniatis, 2013). Epigenetic deregulation of this locus in humans has been implicated in numerous diseases and disorders, including following PAE and ELS (El Hajj et al., 2017). Our group was the first to identify a PAE-associated profile of DNA promoter hypermethylation at the clustered protocadherin locus, which was observed in both the brains of adult mice following PAE *via* continuous preference drinking, as well as the buccal swabs of children born with FASD (Laufer et al., 2015). This result has been replicated in the saliva of children with FASD (Portales-Casamar et al., 2016; Lussier et al., 2018b). However, these studies have noted variation in the specific differentially methylated CpG sites within the locus. This observed variation in single CpG sites across experiments is in line with the stochastic nature of methylation establishment at this locus (Toyoda et al., 2014; Canzio et al., 2019) and highlights that this profile is apparent the level of larger differentially methylated regions rather than single CpG sites. Other factors, such as genetic background, sex, and age, may also contribute to this profile. Additionally, the differences between studies may be related to the nature of cellular content differences, as either buccal epithelial swabs (Laufer et al., 2015) or saliva were assayed (Portales-Casamar et al., 2016; Lussier et al., 2018b). Both sample sources are heterogeneous in that they contain epithelial cells and blood leukocytes, however, buccal swabs have a higher proportion of epithelial cells than saliva samples (Theda et al., 2018). This difference in the cellular composition of oral sample sources may explain some of the differences between studies, particularly when considering the mechanism that results in clustered protocadherin hypermethylation. We hypothesize that the clustered protocadherin hypermethylation profile observed in oral sample sources is from buccal epithelial cells given that both buccal epithelial and brain cells are derived from the ectoderm during development, which creates the potential for buccal epithelial cells to contain epigenetic “footprints” of early neurodevelopmental disruptions (Smith et al., 2015). This hypothesis is supported by our observations in the hippocampus of adult mice following binge ethanol exposure in the trimester three equivalent period. Following PAE, promoters in the clustered protocadherin locus are hypermethylated and have decreased levels of H3K4me3 and H3K27me3 (Chater-Diehl et al., 2016). This finding is notable given that these two histone PTMs make up bivalent chromatin, which plays a key role in development (Bernstein et al., 2006). Together, these results suggest a putative mechanism, where bivalent chromatin is not properly established during neurodevelopment. As a result, DNA methyltransferase 3L (DNMT3L) can recognize the unmodified histone H3 (H3K4me0) and stimulate DNA methyltransferase 3A (DNMT3A) to methylate the locus (Ooi et al., 2007; Otani et al., 2009; Zhang et al., 2010). This action is expected to decrease gene expression (Figure 1), which is a general trend observed across diverse exposure paradigms. This includes from adult mouse whole brain following a trimester 3 equivalent binge-like exposure (Kleiber et al., 2013), and more recently in adult mouse hippocampus following continuous PAE (Alberry et al., 2020). This is exemplified in adult mouse brains from our continuous preference drinking paradigm, where PAE resulted in increased methylation (*p* < 0.01) at the *Pcdhb2* promoter and a corresponding decrease in gene expression (fold change = −1.23, *p* < 0.05; Laufer et al., 2013).

The clustered protocadherins have also been implicated in ELS, where a comparison of the hippocampal tissue from individuals that have experienced abuse as children and rats that received low levels of maternal care revealed DNA promoter hypermethylation and gene body DNA hypomethylation at the locus, corresponding with a repressive effect (Suderman et al., 2012). An integrative analysis of this broad signature of differential DNA methylation in adult hippocampal tissue from rats revealed that low levels of maternal care were also associated with H3K9ac (a histone PTM associated with transcriptional activation) and gene expression at the clustered protocadherins (McGowan et al., 2011).

In an FASD model that includes PAE and ELS in adult mouse hippocampus both hyper- and hypo-methylation in the protocadherin region as well as decreased gene expression resulted from PAE (Alberry and Singh, 2020; Alberry et al., 2020). ELS alone and the combination of PAE and ELS resulted in hypermethylation of the locus and decreased expression of a transcript (Alberry and Singh, 2020; Alberry et al., 2020). Interestingly, transcripts from this locus were within a gene co-expression module that correlates with treatment and anxiety-like behavior (Alberry et al., 2020). Taken together, these results suggest that the clustered protocadherins are sensitive to PAE, ELS, and the cumulative impact of the two treatments. Ultimately, the alterations to epigenomic and transcriptomic profiles of the clustered protocadherin locus likely lead to changes in neuronal identity and synaptic connections, as well as disrupt neural circuits during neurodevelopment.

## Oxidative Stress Pathways in FASD and ELS

Oxidative stress is a well-characterized part of FASD etiology. At the cellular level, ethanol acts directly on mitochondria to produce superoxide, hydroxide, and nitric oxide radicals (Wu and Cederbaum, 2003). Metabolism of ethanol produces oxidized products (Mansouri et al., 2001) and acetaldehyde in the brain, further increasing the formation of reactive oxygen species (Shaw, 1989). Oxidative damage can lead to blood-brain barrier impairment, inflammation, and increased apoptosis (Haorah et al., 2008), key features of FASD etiology (Barak et al., 1996). Importantly, increased oxidative stress is also a key feature of ELS. Studies have found altered oxidative stress in adult male mice following early maternal separation (Malcon et al., 2020) and in plasma of human adolescents reporting childhood maltreatment (do Prado et al., 2016). Lasting oxidative damage to the brain occurs in mice exposed to maternal separation (Réus et al., 2017). Interestingly, treatment with voluntary exercise in adolescence leads to some recovery of mitochondrial function after maternal separation in rats (Sahafi et al., 2018). At the gene expression and epigenetic levels, oxidative stress genes are often implicated after PAE. We found altered expression, DNA methylation, and histone PTMs at oxidative stress and free radical scavenging genes in adult mouse hippocampus following trimester three equivalent ethanol exposure (Chater-Diehl et al., 2016). Other groups have also identified similar results. An integrative analysis of DNA methylation, miRNA expression, and gene expression from rats that received augmented maternal care demonstrated that key oxidative stress genes were affected (Vogel Ciernia et al., 2018). Taken together, these studies show that oxidative stress is a key component of both PAE and ELS, which is also reflected in transcriptomic and epigenomic studies. Targeting such mechanisms may be a promising avenue for therapeutic strategies in the future.

## Light at The End of The Tunnel

**Figure 2:**
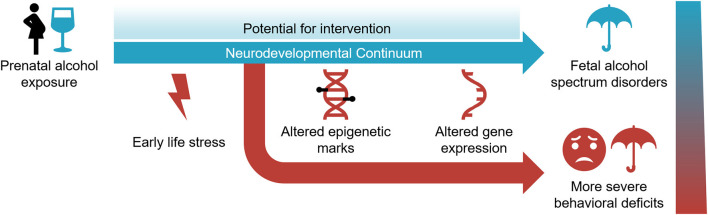
The neurodevelopmental continuum is susceptible to PAE and early life stress (ELS) in the development and severity of fetal alcohol spectrum disorder. Epigenetic modifications and gene expression are affected by these environmental exposures. While it represents a sensitive period for environmental assaults, the dynamic nature of the neurodevelopmental continuum also offers potential for intervention.

FASD is a common neurodevelopmental disorder that represents a societal burden with no effective treatments. Yet, it is entirely preventable. It requires the avoidance of alcohol before and during pregnancy. This is a challenging proposition given that the use of alcohol in reproductive-age women is increasing and many pregnancies are unintended, which is expected to lead to a corresponding increase in FASD (Alberry and Singh, 2019). Under the circumstances, there is a need to better understand the molecular mechanisms involved and develop novel treatment modalities. The research discussed in this review argues that multiple epigenetic mechanisms are involved in the initiation, progression, and maintenance of aberrant phenotypes associated with FASD. Epigenetic mechanisms are dynamic and operational during the neurodevelopmental continuum spanning prenatal and postnatal periods (Figure 2). It offers the potential to reverse the outcome *via* epigenetic manipulation.

To this end, research regarding the prevention of FASD has centered on increasing the availability of methyl donors during development through supplementation of known methyl donors such as folate and choline. In an embryonic cell culture model, co-administration of folic acid prevents the morphological delays and deficits, together with the downregulation of *Hoxa1* and its miRNA, *miR-10a* (Wang et al., 2009). Where PAE results in malformations, offspring of pregnant mice on a diet supplemented with choline, betaine, folic acid, vitamin B12, L-methionine, and zinc, followed by PAE have fewer malformations than those with PAE alone (Downing et al., 2011). Gestational choline supplementation in a rat model reduces ethanol-associated alterations in gene expression and protein levels of DNA methylation genes *Dnmt1* and *Mecp2* in the hypothalamus (Bekdash et al., 2013). Similarly, choline supplementation prevents the ethanol-associated changes in gene expression of histone methylation genes *Set7/9*, *G9a*, and *Setdb1*, as well as the detected changes in histone PTMs, H3K4me2, 3 and H3K9me2 (Bekdash et al., 2013). Also, choline supplementation blocks the ethanol-associated increase in *POMC* promoter methylation and corresponding decreased gene expression in the hypothalamus (Bekdash et al., 2013). Betaine supplementation during embryonic ethanol exposure in an avian embryo model reduces malformation defects observed following ethanol alone (Karunamuni et al., 2017). Following PAE, choline supplementation and working memory training in adolescent rats sufficiently improve the cognitive flexibility and connectivity deficits measured in adulthood (Waddell et al., 2020). Choline supplementation as an intervention for women drinking during pregnancy has been minimally investigated. However, choline-supplemented infants have improved weight and head circumference at 6.5 and 12 months, in addition to better visual recognition memory compared to placebo infants (Jacobson et al., 2018). In a trial of choline administration in 2.5- to 5-year-olds with FASD, children who receive 500 mg of choline daily for 9 months have higher non-verbal intelligence, visual-spatial skills, working memory, verbal memory, and fewer symptoms associated with ADHD than the placebo group when assessed 4 years later (Wozniak et al., 2020). While the methyl-donor supplementation hypothesis has gained support, the precise mechanism of action is still unknown. Still, it remains an exciting avenue for potential prevention of FASD-related deficits following PAE. However, it remains an exciting avenue for potential prevention of FASD-related deficits following PAE. Further understanding of how the postnatal environment may modulate FASD-related deficits will come from animal models. In one study, decreased dendritic complexity in the hippocampus associated with binge-like trimester three-equivalent exposure was avoided by exercise and environmental complexity (Boschen et al., 2017). Such results on epigenetic interventions are encouraging for most neurodevelopmental disorders that result from environmental exposures. This optimism must be tempered with the observation that for most of these studies, a few metrics show improvement but many outcomes of PAE remain. This is likely due to structural changes occurring in the brain following neuronal apoptosis that are not possible to rectify by any manner of available postnatal treatments. This is not to negate the positive outcomes of these studies but should focus our attention on the prenatal fetal environment for treatments during this time.

Further consideration should also be paid to the types of experiments used in FASD research. To date, most studies have used bulk tissue or whole embryo inputs for molecular experiments. These have provided many valuable insights to this point, however, given the heterogeneity of the effects of PAE and preferential apoptosis of specific cell types, more focused studies are needed. Specifically, single-cell epigenetic and transcriptomic techniques are now viable for most FASD models. Single-cell or single-cell-type resolution may address many outstanding questions about the outcomes of ethanol exposure at the molecular level. Furthermore, single-cell studies and improved bioinformatic cellular deconvolution methods will aid future translational studies examining the DNA methylation signature of FASD in saliva (Turinsky et al., 2019) and other non-invasively obtained samples.

## Conclusion

Neurodevelopment in mammals is a long-lasting, highly orchestrated, and coordinated continuum. It is highly sensitive and responsive to environmental exposures that may determine the outcome and resulting brain function, including neurodevelopmental disorders such as FASD. FASD is highly heterogeneous and a direct result of maternal alcohol consumption during pregnancy. Further, a stressful postnatal environment may worsen the outcomes. This review emphasizes the role of dynamic epigenetic changes during the neurodevelopmental continuum, from prenatal to postnatal stages in the development of FASD, including ncRNAs, DNA methylation, and histone PTMs. We argue that FASD is an epigenetic disorder. Additionally, the early postnatal environment may determine the severity of the effect of PAE. It may be possible to moderate this effect by postnatal epigenetic modifications, which may or may not be driven by pharmaceutical interventions. This perspective offers new hope for the development of effective treatment for FASD. Finally, the strategy of epigenetic intervention discussed here may apply to a variety of neurodevelopmental disorders involving epigenetic marks caused by environmental exposures.

## Author Contributions

All authors contributed to the design and organization of this review. All authors performed a literature review, as well as interpretation and synthesis of findings. BA created the first draft, with all authors substantially contributing to revisions. All authors have approved the final revisions prior to submission. All authors contributed to the article and approved the submitted version.

## Conflict of Interest

The authors declare that the research was conducted in the absence of any commercial or financial relationships that could be construed as a potential conflict of interest.
